# Leveraging Attention-Based Deep Learning in Binary Classification for Early-Stage Breast Cancer Diagnosis

**DOI:** 10.3390/diagnostics15060718

**Published:** 2025-03-13

**Authors:** Lama A. Aldakhil, Shuaa S. Alharbi, Abdulrahman Aloraini, Haifa F. Alhasson

**Affiliations:** Department of Information Technology, College of Computer, Qassim University, Buraydah 52571, Saudi Arabia; 441212544@qu.edu.sa (L.A.A.); shuaa.s.alharbi@qu.edu.sa (S.S.A.); a.aloraini@qu.edu.sa (A.A.)

**Keywords:** convolutional neural networks, deep learning, machine learning, transfer learning, breast cancer, histology images, image classification

## Abstract

**Background:** Breast cancer diagnosis is a global health challenge, requiring innovative methods to improve early detection accuracy and efficiency. This study investigates the integration of attention-based deep learning models with traditional machine learning (ML) methods to classify histopathological breast cancer images. Specifically, the Efficient Channel-Spatial Attention Network (ECSAnet) is utilized, optimized for binary classification by leveraging advanced attention mechanisms to enhance feature extraction across spatial and channel dimensions. **Methods:** Experiments were conducted using the BreakHis dataset, which includes histopathological images of breast tumors categorized as benign or malignant across four magnification levels: 40×, 100×, 200×, and 400×. ECSAnet was evaluated independently and in combination with traditional ML models, such as Decision Trees and Logistic Regression. The study also analyzed the impact of magnification levels on classification accuracy, robustness, and generalization. **Results:** Lower magnification levels consistently outperformed higher magnifications in terms of accuracy, robustness, and generalization, particularly for binary classification tasks. Additionally, combining ECSAnet with traditional ML models improved classification performance, especially at lower magnifications. These findings highlight the diagnostic strengths of attention-based models and the importance of aligning magnification levels with diagnostic objectives. **Conclusions:** This study demonstrates the potential of attention-based deep learning models, such as ECSAnet, to improve breast cancer diagnostics when integrated with traditional ML methods. The findings emphasize the diagnostic utility of lower magnifications and provide a foundation for future research into hybrid architectures and multimodal approaches to further enhance breast cancer diagnosis.

## 1. Introduction

The rising global incidence of breast cancer (BC) has become a major public health concern, but early detection remains critical in significantly improving survival rates and patient outcomes. Various imaging modalities play a crucial role in identifying BC at its early stages. These images including mammography for abnormalities, ultrasound for dense tissue evaluation, MRI for detailed imaging in high-risk cases, and histopathology for microscopic cancer analysis. Together, they improve early diagnosis and patient outcomes.

The diagnosis of BC tumor tissues is a challenging process influenced by numerous factors. It demands a high level of expertise and meticulous attention to detail to differentiate between various breast tumor types, making it both complex and time-consuming. Deep learning (DL) approaches have demonstrated significant potential as assistive diagnostic tools for BC tumors, addressing these challenges effectively. In recent years, advancements in deep learning have introduced attention mechanisms, which represent a notable innovation beyond traditional convolution operations. Attention mechanisms function as a refined step in image analysis, assigning critical facets of an image to specific classes and enhancing the model’s focus on essential components. These mechanisms improve the precision and accuracy of tasks by selectively concentrating on the most relevant features of an image, akin to the way humans focus on key facial features for recognition.

In deep learning models, attention mechanisms play a pivotal role by prioritizing significant elements, much like the parsing strategies used in natural language processing. This selective focus enhances the interpretive capabilities of models, yielding better results and more human-like performance in visual tasks. Recent studies, such as those made by Dosovitskiy et al. [1] with Vision Transformer (ViT) and Vaswani et al. [2] with the Transformer model, have demonstrated the effectiveness of attention mechanisms in improving performance across visual and sequential tasks. Models like ViT and DETR [3] further show how attention mechanisms allow models to focus on specific regions of an image, improving object detection and overall task performance. We will also update the Introduction section to clarify this rationale and emphasize the role of attention mechanisms in the proposed approach.

However, the accurate diagnosis of tumors necessitates the meticulous examination of tissue samples by experienced pathologists. This diagnostic procedure poses significant challenges due to its inherent difficulty and time-consuming nature. BC histopathology slide images exhibit complex features that require thorough examination. Introducing a deep learning (DL) approach as an assistive tool for pathologists can significantly enhance their workflow, reduce error margins, and expedite the diagnostic process.

Although many deep learning-based approaches have been proposed in the literature, relatively few have integrated attention mechanisms. Attention mechanisms enable CNNs models to improve their representational power by focusing on salient features, thereby enhancing their generalization capabilities [4]. The method, referred to as the Efficient Channel-Spatial Attention Network (ECSAnet) [5], incorporates a convolutional block attention module (CBAM) within its architecture. This integration enables ECSAnet to proficiently extract information from both channel and spatial dimensions. It optimizes the representation of features by isolating the most relevant ones, concentrating on their importance and precise position within the image.

In BC histopathology, tumor condition analysis begins with histopathological slides being viewed under varying levels of magnification. The four most frequently used levels are referred to as 40×, 100×, 200×, and 100× magnifications. A pathologist using a light microscope first examines the breast tissue sample at a lower magnification, such as 100×, to study the overall distribution of nuclei and tissue structure, which helps to identify areas of interest and determine whether cells and tissue are normal or abnormal. The pathologist then switches to a higher magnification, such as 100×, to analyze cellular morphology in greater detail, focusing on features like nuclear structure and the number of mitotic figures. The amount and appearance of mitotic figures in the pathologist’s report are significant factors in determining the grade of breast cancer. According to Rashmi et al. [6], prioritizing the analysis of 40× and 100× magnification images is recommended to enhance the accuracy of breast cancer diagnosis.

Therefore, the choice of magnification often depends on the specific diagnostic protocol and the type of tissue being examined. For instance, low magnifications might be preferred when distinguishing between benign and malignant lesions, while higher magnifications are necessary for identifying subtle cellular abnormalities and specific cancer subtypes. Additionally, digital pathology and image analysis software can enhance traditional methods by allowing pathologists to zoom in and out seamlessly, combining insights from multiple magnifications for comprehensive assessments. The integration of AI-assisted tools is also becoming increasingly common, aiding in the identification of features across various magnifications [7].

The primary contribution of this study lies in improving the accuracy of DL-based breast cancer diagnosis by incorporating machine learning (ML) techniques for binary classification, distinguishing between benign and malignant cases using different magnification factors, with a focus on early-stage detection. The decision to use a DL-to-ML pipeline rather than a fully DL model stems from the complementary strengths of both approaches. Fully DL models, while powerful, often require extensive computational resources and large datasets, which can be limiting in histopathological applications. By leveraging ECSAnet for robust feature extraction and combining it with traditional ML classifiers (e.g., Logistic Regression or Decision Trees), the proposed approach achieves better flexibility, interpretability, and computational efficiency. This hybrid method also performs well on smaller datasets like BreakHis, where traditional ML classifiers can effectively utilize extracted features, making it a practical solution for breast cancer diagnostics.

The structure of this paper is as follows: Section 2 provides a review of related work relevant to this research. Section 3 describes the core aspects of the applied methodology. Section 4 presents the results obtained, followed by a discussion of these findings. Finally, Section 6 outlines potential directions for future research and concludes the study.

## 2. Related Work

In pursuing BC classification, Gupta et al. [8] employed features extracted using pre-trained CNNs, specifically VGG16, VGG19, Xception, and ResNet50. The extracted features were subsequently utilized in two distinct ML approaches: Support Vector Machines (SVM) and Logistic Regression (LR). Two models were trained for both SVM and LR. One model considered the magnification level as a dependent variable, while the other did not. The results revealed that the combination of ResNet50 with LR achieved a maximum accuracy of 93.27%, whereas the combination of ResNet50 with SVM attained a maximum accuracy of 92.5%.

Murtaza et al. [9] presented BMIC_net, a BC multi-classification network known as the Biopsy Microscopic Image Cancer Network. BMIC_net is built upon a pre-trained AlexNet and consists of an initial AlexNet-like segment followed by fine-tuned, fully connected layers. The output layer of BMIC_net is customized to accommodate the histopathology image classes in the dataset. The classification process in BMIC_net follows a hierarchical model with two levels. At the first level, binary classification determines whether the tissue is benign or malignant. The second level employs separate classifiers for benign and malignant cases, enabling classification into subtype categories. The benign classifier identifies subtypes within benign images, while the malignant classifier does the same for malignant images. To determine the optimal ML model for each classification level, six models were evaluated: K-Nearest Neighbor (KNN), SVM, Naive Bayes (NB), LR, Decision Trees (DT), and Linear Discriminant Analysis (LDA). Due to the large feature sizes extracted by the model, feature reduction techniques such as information gain (IG) and principal component analysis (PCA) were employed.The experimental results revealed that IG outperformed PCA in selecting the most discriminative features. Moreover, KNN exhibited superior performance compared to other ML models, achieving accuracies of 95.48%, 94.63%, and 92.45% for binary, benign, and malignant classification, respectively.

Gour et al. [10] presented ResHist, a novel breast histology image classification method based on the ResNet50 architecture. ResHist extends ResNet50 by increasing the number of layers to 152, enabling the model to capture more intricate and representative features at various levels of abstraction. The experimental results demonstrated the effectiveness of ResHist, achieving classification accuracies ranging from 87.4% to 92.52%. Furthermore, ResHist was evaluated as a feature descriptor for four classifiers: Random Forest (RF), Quadratic Discriminant Analysis (QDA), SVM, and KNN. Among these classifiers, the SVM classifier performed the best, yielding an accuracy of 92.42%. However, it is worth noting that the increased number of layers in ResHist resulted in higher computational complexity, which represents a trade-off for a modest 0.50% improvement in accuracy over the original ResNet50 model.

Ahmad et al. [11] proposed three transfer learning (TL)-based approaches for BC classification from histopathological images, utilizing the EfficientNet architecture. First, image-wise classification was performed by fine-tuning pre-trained EfficientNet models (B0, B2, B5, and B7). Second, deep features extracted from the EfficientNet models were utilized in an SVM model for classification. Third, discriminative patches were identified through dendrogram clustering at different magnification levels. These selected patches were then normalized using Z-score normalization and fed into the EfficientNet models for classification. The results were remarkable across different magnification levels, demonstrating high accuracies ranging from 78% to 99.99% for patch-wise, image-wise binary, and multi-class classification.

Clement et al. [12] introduced a multi-scale pooled image feature representation (MPIFR) architecture for extracting BC features. Their approach implemented an ensemble of four pre-trained deep convolutional neural networks (DCNNs): ResNet50, ResNet18, DenseNet201, and EfficientNetB0, which were selected based on superior performance. The extracted features were fed into a one-vs-one SVM to enable eight-class multi-classification across four magnification levels. The proposed method achieved an average accuracy of 97.77%.

Most recently, Aldakhil et al. [5] introduced an attention-based deep learning model for the multi-classification of histopathological images of breast cancer, employing the Efficient Channel-Spatial Attention Network (ECSAnet). This attention mechanism enhances the identification and emphasis of crucial regions in the images. By combining EfficientNetV2 with attention layers, the model adeptly distinguishes between various subtypes of breast cancer. Validation metrics demonstrated the performance level of this system. ECSAnet’s performance was assessed using the BreakHis dataset at four magnification levels (40×, 100×, 200×, and 400×), achieving accuracies ranging from 88.41% to 94.2%, with the highest accuracy observed at 400×. The system showed improvement across various performance metrics, including accuracy, precision, recall, and the F1-score. These results suggest that ECSAnet is a robust and reliable tool with significant potential to assist diagnostic pathologists working in the field of breast cancer histopathology.

While prior works have advanced breast cancer (BC) classification, they exhibit limitations in feature prioritization, handling magnification-specific variability, and effectively integrating deep learning with traditional machine learning (ML). Gupta et al. [8] and Ahmad et al. [11] relied on sequential deep feature extraction and ML classifiers, which underutilize the hierarchical capabilities of CNNs. Similarly, custom architectures like ResHist [10] improved accuracy but at the cost of higher computational complexity. Attention-based models, such as ECSAnet [5], improved feature focus, but lacked an exploration of hybrid approaches or magnification-level performance.

The proposed work addresses these gaps by combining ECSAnet with traditional ML classifiers (e.g., Logistic Regression, Decision Trees), leveraging attention mechanisms to enhance feature extraction while improving performance across all magnification levels. This hybrid approach achieves better accuracy, particularly at higher magnifications, while maintaining computational efficiency. By explicitly aligning diagnostic objectives with magnification variability, the method ensures robust and practical BC classification, paving the way for further advancements in hybrid architectures.

## 3. Methods and Materials

ECSAnet, proposed by Aldakhil et al. [5], is specifically designed to extract discriminative features from histopathology images for breast cancer classification. Built upon the EfficientNetV2-S architecture [13], ECSAnet introduces three key enhancements to improve its performance:

1. Incorporation of the Convolutional Block Attention Module (CBAM): ECSAnet integrates a CBAM before the final classifier block. This module refines spatial and channel-wise features, enhancing the model’s ability to capture subtle variations in histopathological images. This is particularly critical for distinguishing between tumor subtypes and addressing variability in image characteristics.
2. Augmentation of the Final Classifier Block: The final classifier block is enhanced with the addition of two fully connected (FC) layers. These layers improve feature representation and interpretation, ultimately boosting the classification accuracy.
3. Reduction in the Number of Output Features: The number of output features in the last FC layer is reduced from 1000 to 8, aligning the output with the eight tumor subtypes in the BreakHis dataset. This modification ensures that the model is tailored to the specific classification task.

**Figure 1:**
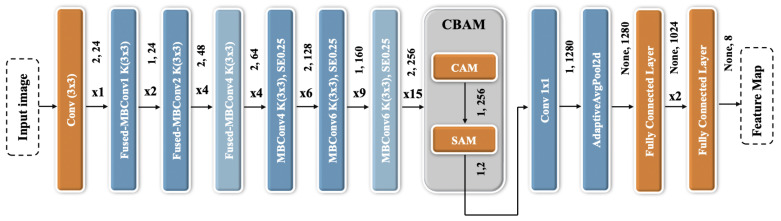
Architecture of ECSAnet [5].

These enhancements enable ECSAnet to effectively address challenges such as variations in magnification levels and tumor heterogeneity, thereby improving its overall effectiveness in breast cancer diagnosis. A summary of the ECSAnet architecture is presented in Figure 1.

### 3.1. Efficient Channel-Spatial Attention Network

The Efficient Channel-Spatial Attention Network (ECSAnet), introduced by Aldakhil et al. [5], builds upon the EfficientNetV2 [13] architecture and incorporates enhancements such as the convolutional block attention module (CBAM) [14] and additional fully connected layers. CBAM efficiently captures features across both the channel and spatial dimensions, refining them by focusing on the most relevant features and emphasizing what is important and where it is located within an image. The final classifier block is improved with two additional fully connected layers, and the output features of the last fully connected layer are reduced from 1000 to 8 to align with the eight tumor subtypes in the BreakHis dataset. These modifications increase the model’s learning capacity and adaptability for this specific classification task. The architecture’s primary components include MBConv [15] and Fused-MBConv [16] layers. MBConv combines a Squeeze-and-Excitation module with a depth-wise convolutional layer, while Fused-MBConv replaces the depth-wise convolution with a standard 3 × 3 convolutional layer, further optimizing performance. Combining attention mechanisms, such as CBAM, with traditional ML classifiers is justified by their complementary strengths. Attention mechanisms enhance feature extraction by focusing on the most relevant regions of an image, improving the representation of critical features, while traditional ML classifiers excel at leveraging these refined features for robust decision-making. This combination addresses the challenges of complex medical image analysis by improving interpretability, refining feature importance, and enhancing classification accuracy, making it a particularly effective approach for such tasks.

### 3.2. Machine Learning Approaches

Different traditional classifiers are used in this study as they are versatile across various tasks and serve as widely recognized benchmarks in machine learning research. Their inclusion ensures that the comparison is meaningful, fair, and aligned with standard evaluation practices. The details of these classifiers can be found here:

#### 3.2.1. Support Vector Machines (SVM)

Support Vector Machines (SVM) is a widely used supervised machine learning algorithm designed for both classification and regression tasks. The primary objective of SVM is to determine the optimal hyperplane that maximizes the separation margin between the nearest data points of different classes. This hyperplane serves as a linear decision boundary, effectively partitioning the feature space into distinct regions representing the respective classes. SVM has been extensively explored in the context of breast cancer classification, as demonstrated by previous studies such as [10,11,12]. The goal is to minimize the following objective function:

(1) $$\begin{array}{c} \underset{w,b,\xi }{\mathrm{minimize}}\;\frac{1}{2}{∥w∥}^{2}+C\sum_{i=1}^{n}\xi_{i} \end{array}$$

Subject to the following constraints:

(2) $$\begin{array}{c} y_{i}\left(w\;\cdot \;x_{i}+b\right)\geq 1-\xi_{i},\;\xi_{i}\geq 0,\;\forall i \end{array}$$

where

w is the weight vector that defines the hyperplane.

*b* is the bias term.

ξ indicates the slack variables that allow for soft-margin classification.

C>0 is the regularization parameter that balances the trade-off between maximizing the margin and minimizing classification errors.

$y_{i}\in \left\{-1,+1\right\}$ represents the class labels.

$x_{i}$ denotes the feature vectors.

#### 3.2.2. K-Nearest Neighbor (KNN)

The K-Nearest Neighbor (KNN) classification algorithm operates on the principle that patterns in close proximity to a target pattern (denoted as $x^{\prime }$) contain valuable information about its label. KNN classifies a data point by assigning it the majority class among its *K* nearest neighbors within the dataset. To achieve this, a similarity measure, such as Euclidean distance, is employed to quantify the closeness of patterns in the data space. The performance of KNN is influenced by the choice of *K*, which determines the size of the neighborhood considered for classification, thereby defining the algorithm’s locality [17]. This is shown as follows:

(3) $$\begin{array}{c} k\left(x,x^{\prime }\right)=\sqrt{\sum_{i=1}^{n}{\left(x_{i}-x_{i}^{\prime }\right)}^{2}} \end{array}$$

#### 3.2.3. Linear Discriminant Analysis (LDA)

Linear Discriminant Analysis (LDA) is a widely used technique for data classification and dimensionality reduction. Its primary objective is to improve class separability by maximizing the ratio of between-class variance to within-class variance in the dataset. This optimization facilitates effective classification, even when class frequencies are imbalanced. LDA employs two distinct strategies: class-dependent transformation, which maximizes the ratio of between-class variance to within-class variance for each class individually, and class-independent transformation, which optimizes the overall ratio of total between-class variance to within-class variance across all classes [18]. LDA can be expressed mathematically as follows:

(4) $$\begin{array}{c} J\left(w\right)=\frac{w^{T}S_{B}w}{w^{T}S_{W}w} \end{array}$$

where $S_{B}$ and $S_{W}$ are the between-class and within-class scatter matrices, respectively.

#### 3.2.4. Decision Trees

Decision Trees (DT) are versatile classifiers capable of handling both nominal and numerical attributes. A DT can be visualized as a hierarchical tree structure composed of three main components: a root node, internal nodes, and leaf nodes. The root and internal nodes represent attributes, while the branches correspond to decision rules associated with those attributes. The classification process involves traversing the tree from the root node to a leaf node, guided by the outcomes of attribute-based tests at each internal node. The final leaf node represents the predicted class label for the input instance [19]. This is shown as follows:

(5) $$\begin{array}{c} H=-\sum_{i=1}^{n}p_{i}\log\left(p_{i}\right) \end{array}$$

where

*H* is the entropy, which quantifies the amount of uncertainty or disorder in the system or dataset.

$p_{i}$ probability of the i-th event or class. This represents the proportion of instances belonging to class *i* in the dataset.

*n* is the number of distinct events or classes.

#### 3.2.5. Naïve Bayes

The Naïve Bayes (NB) classifier is a widely used method in image classification research due to its competitive accuracy and computational efficiency. It is based on Bayes’ theorem and operates under the assumption that the features (attributes) of a dataset are conditionally independent, given the class label. The NB classifier utilizes information from the training dataset to compute the posterior probability P(y|x) for each class *y*, given an input instance *x* [20]. Bayes’ theorem is expressed as follows:

(6) $$\begin{array}{c} P\left(y|x\right)=\frac{P(y)P(x|y)}{P(x)} \end{array}$$

where

P(y|x) is the posterior probability of class *y* given the input *x*.

P(y) is the prior probability of class *y*.

P(x|y) is the likelihood of *x* a given class *y*.

P(x) is the marginal probability of the input *x*.

The Naïve Bayes classifier predicts the class with the highest posterior probability, making it a simple yet effective approach for classification tasks.

#### 3.2.6. Logistic Regression

Logistic Regression (LR), specifically multinomial LR, is a statistical classification method used when the dependent variables in the dataset are categorical and have more than two classes. In multinomial LR, the first step involves calculating each class’s logits (log odds). This is performed by taking the linear combination of the input variables and their corresponding coefficient values using a logit function. Subsequently, a softmax function is applied to the logits to obtain the predicted probabilities for each class [21].

(7) $$\begin{array}{c} P\left(y=1|X\right)=\sigma \left(w^{T}X+b\right)=\frac{1}{1+e^{-(w^{T}X+b)}} \end{array}$$

where P(y=1|X) is probability that the output *y* is equal to 1, given the input feature vector *X*.

### 3.3. Proposed Framework

**Figure 2:**
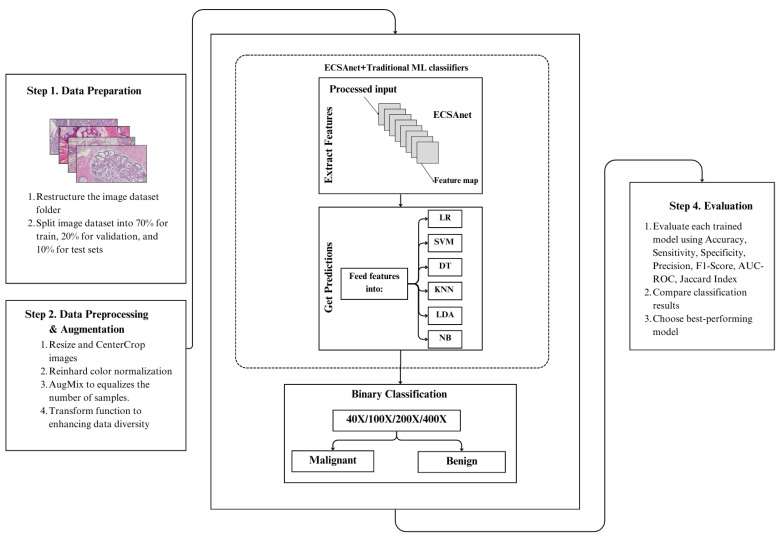
The workflow of the proposed approach. Our approach starts with dataset preprocessing, proceeds to training the ECSAnet model for feature extraction and classification predictions, and concludes with model enhancement by ML models and evaluation steps.

This work is an optimization of ECSAnet proposed by Aldakhil et al. [5], which demonstrated the effect of ECSAnet on a multi-class classification of the BreakHis dataset. Our target is to enhance the ECSAnet results for binary classification, including benign and malignant, across different magnifications, aiming to help in identifying the early stages of diagnosis. In order to enhance the results of ECSAnet, we examine the effect of different ML approaches, including DT, KNN, LDA, LR, NB, and SVM. Figure 2 shows the workflow of the proposed approach.

### 3.4. Dataset

**Figure 3:**
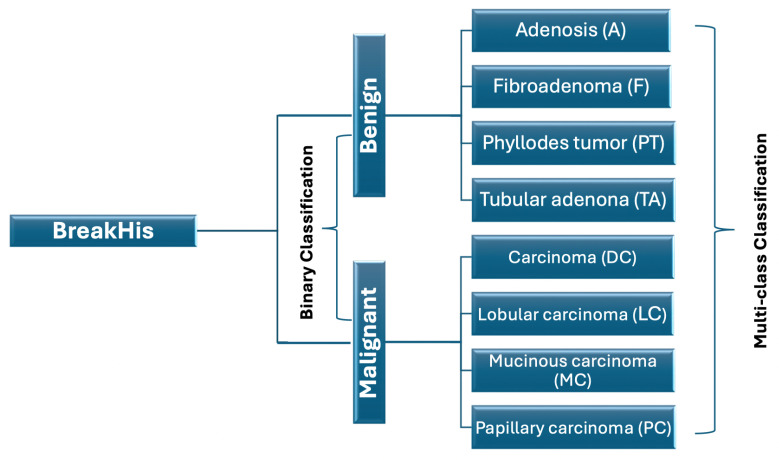
BreakHis classification scheme [22]. Where the Benign class includes different subclasses: Adenosis (A), Fibroadenoma (F), Phyllodes Tumor (PT), and Tubular Adenona (TA). The Malignant class includes different subclasses: Carcinoma (DC), Lobular Carcinoma (LC), Mucinous Carcinom (MC), and Papillary Carcinoma (PC).

The BreakHis dataset is a comprehensive collection of breast cancer histopathological images, specifically designed to facilitate the development and evaluation of machine learning models for cancer classification tasks. It comprises 7909 microscopic images of breast tumor tissue collected from 82 patients, all of whom underwent procedures to excise either benign or malignant breast tumors. Figure 3 illustrates the BreakHis classification scheme, which categorizes breast histopathological images into two primary classes: benign and malignant, each further subdivided into specific subtypes. The benign category includes adenosis, fibroadenoma, phyllodes tumor, and tubular adenoma, while the malignant category comprises ductal carcinoma, lobular carcinoma, mucinous carcinoma, and papillary carcinoma. The images were captured at four different magnification levels—40×, 100×, 200×, and 100×—and are carefully annotated and classified to ensure accuracy and reliability.

**Figure 4:**
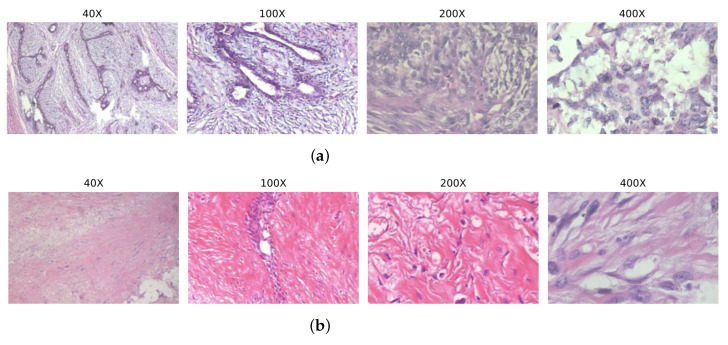
Sample images from BreakHis Dataset [22], where (**a**) shows benign tumor tissues and (**b**) malignant tumor tissues.

Figure 4 illustrates sample images from the dataset, showcasing two classes: the Benign Fibroadenoma (F) class and the Malignant Lobular Carcinoma (LC) class. These examples highlight the diversity of tumor morphology under varying magnifications, which is a critical aspect of the dataset.

The BreakHis dataset is publicly available and can be accessed online at the following link: Breast Cancer Histopathological Database (BreakHis) (https://web.inf.ufpr.br/vri/databases/breast-cancer-histopathological-database-breakhis/, accessed on 21 February 2025).

#### 3.4.1. Data Analysis

**Figure 5:**
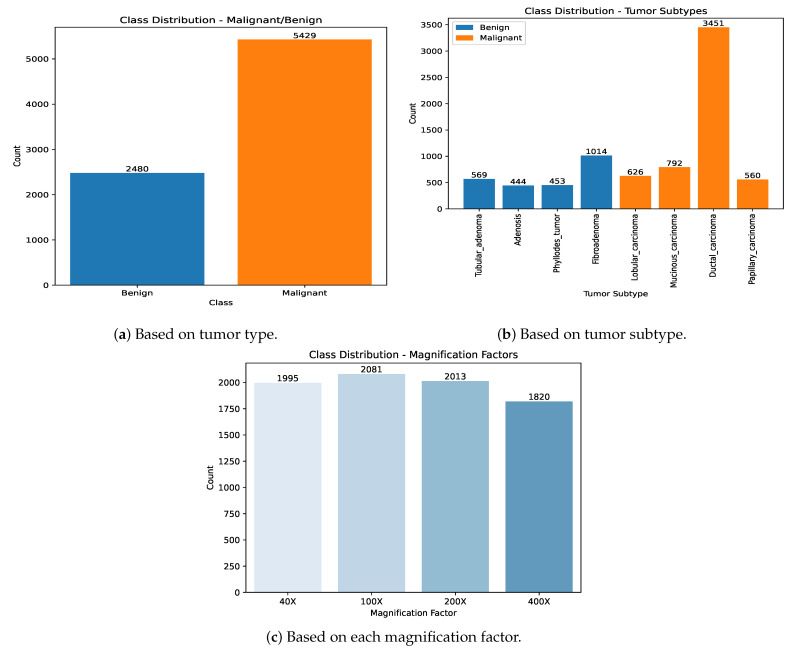
Visual analysis of the BreakHis dataset.

To extract meaningful insights from a dataset, visualizing its data distribution is essential. Figure 5a–c present the class distribution of samples within the BreakHis dataset. Analyzing these distributions highlights significant patterns and imbalances, as further illustrated in Figure 5. The key observations derived from this analysis are as follows:

1. Class Imbalance Between Benign and Malignant Samples: The malignant class significantly outnumbers the benign class, revealing a substantial disparity in sample sizes.
2. Prevalence of Ductal Carcinoma (DC): Within the eight-class distribution, samples belonging to the Ductal Carcinoma (DC) category are notably more frequent compared to other tumor subtypes.
3. Balanced Distribution Across Magnification Levels: The dataset exhibits a relatively uniform distribution of samples across the four magnification factors (40×, 100×, 200×, and 100×).

These observations emphasize the importance of addressing class imbalance during model training to ensure fair and effective classification performance. Additionally, the consistent distribution across magnification factors provides an opportunity to explore multi-scale feature extraction techniques.

Class imbalances in datasets pose significant challenges for training ML models. They often result in misleading accuracy metrics, biased performance favoring the majority classes, and reduced generalization capabilities. Furthermore, inconsistencies in H&E staining procedures across different pathology laboratories can exacerbate these issues, further limiting the model’s ability to generalize effectively. To mitigate these challenges and improve the overall quality of the dataset, the subsequent sections will outline strategies to address class imbalances and propose standardization procedures for H&E staining methodologies.

#### 3.4.2. Data Preprocessing and Augmentation

**Figure 6:**
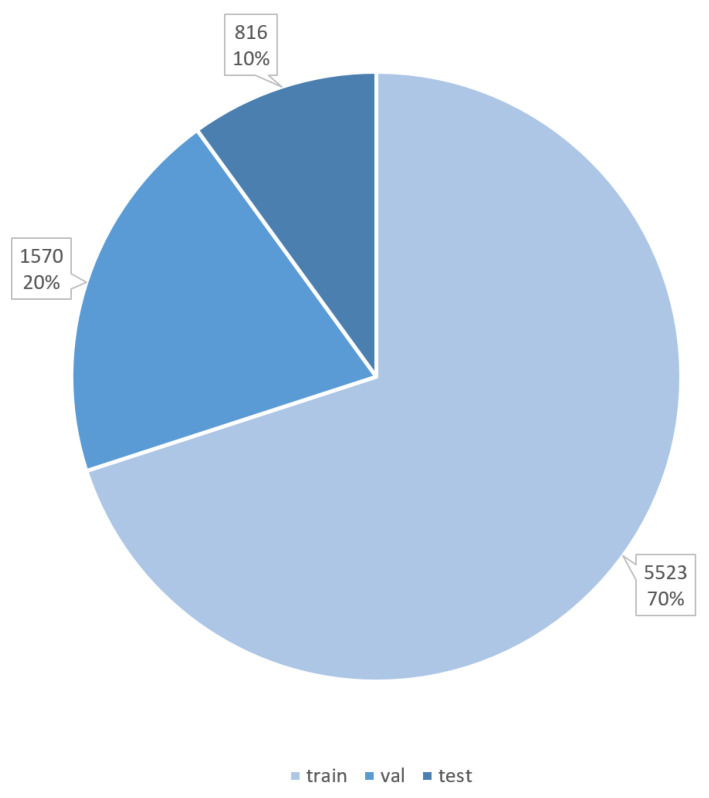
Dataset after splitting into training, validation, and test sets.

**Figure 7:**
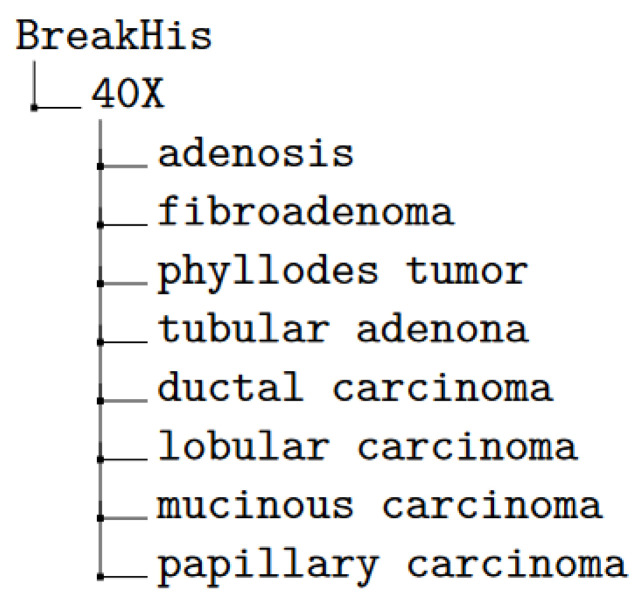
Directory tree of the reorganized dataset folder structure.

To prepare the BreakHis dataset for model training, its organizational structure was adjusted to support both multi-class and binary classification tasks. Four primary folders corresponding to the magnification levels (40×, 100×, 200×, and 100×) were created, allowing models to account for magnification factors during training. Within each folder, subfolders were organized for the eight breast tumor subtypes, ensuring a clear and hierarchical structure. This reorganization facilitated image-level classification while disregarding patient associations. The dataset was then partitioned into three segments: 70% for training (5523 images), 20% for validation (1570 images), and 10% for testing (816 images), as illustrated in Figure 6. A visual representation of the folder structure is shown in Figure 7.

To standardize input data and ensure consistency, image normalization was applied. The pixel values in each channel were adjusted to specific mean and standard deviation values—[0.485, 0.456, 0.406] and [0.229, 0.224, 0.225], respectively. All images were resized and cropped to uniform dimensions of 384 × 384 pixels. Additionally, the Reinhard color normalization method [23] was used to reduce variations caused by differences in histopathological staining protocols. This method adjusted the color distribution of images to match a predefined reference set, ensuring consistency across the dataset. The decision not to normalize to a mean of zero and a standard deviation of one was based on the specific nature of the features in the histopathological images. Instead, normalization values derived from ImageNet statistics were used, aligning with pre-trained deep learning models to ensure better feature representation.

To address class imbalance and improve dataset diversity, we applied a two-step data augmentation pipeline, as described in previous work [5]. This approach combines balancing and oversampling strategies:

- Balancing: The AugMix technique [24] was applied exclusively to the minority classes to balance the dataset. AugMix generates diverse augmented images by blending multiple transformations (e.g., rotations, posterizations, and distortions). Blending weights were drawn from a Dirichlet distribution, ensuring diversity without excessive duplication.
- Oversampling: After balancing, geometric transformations were applied to all classes to oversample the entire dataset to three times its original size. These transformations included random horizontal and vertical flips (each with a 50% probability), random rotations (affine transformations with angles from −45 to 45 degrees), random translations (up to 10% of the image dimensions), random scaling (with factors between 0.8 and 1.2), and random shearing (with angles from 0 to 10 degrees). This step enriched the dataset with realistic variations to improve model generalization.

The balancing step specifically addressed class imbalance by oversampling minority classes, achieving an even class distribution. In contrast, the oversampling step introduced augmentations across all classes, enhancing diversity and enabling the model to generalize effectively to unseen data. Together, these methods mitigated class imbalance and improved dataset diversity. Table 1 summarizes the class distribution in the BreakHis dataset, and Table 2 illustrates the training data distribution before and after balancing and augmentation.

### 3.5. Experiments Setup and Evaluation Metrics

The technical implementation of the proposed approach leverages Google Colab Pro as a cloud-based development environment, offering access to advanced hardware resources such as GPUs and TPUs. This ensures efficient training and evaluation of deep learning models. Additionally, Google Drive is utilized as a cloud-based storage solution, enabling seamless dataset management and access. This setup not only ensures scalability and ease of use but also facilitates the replication and extension of the work, even in resource-constrained environments.

To evaluate the performance of the proposed method, various metrics were employed, including precision (positive predictive value), recall (sensitivity), the Jaccard index, and the F1-score. These metrics provide a comprehensive assessment of the model’s effectiveness in the classification and analysis tasks. The definitions and corresponding formulas for these metrics are summarized in Table 3.

The success of training a deep learning model is highly dependent on the appropriate selection of hyperparameters. Table 4 outlines the hyperparameter values used in this study. Key parameters, including the learning rate, optimizer, loss function, and early stopping criteria, were meticulously tuned to achieve optimal model performance.

For the machine learning models, all features extracted using the ECSAnet model [5] were standardized using a standard scaler. Additionally, hyperparameter selection was conducted through a grid search across various combinations. The results demonstrated that the default settings in the scikit-learn library performed comparably to or better than the tuned hyperparameter configurations. Consequently, the default settings were adopted to maintain consistency and simplify the evaluation process.

## 4. Experimental Results and Discussion

This section will provide a detailed presentation of the quantitative results across magnification factors.

### 4.1. Quantitative Results

The results in Table 5 comprehensively compare the performance of ECSAnet [5] as a standalone model and in hybrid configurations with traditional ML classifiers across four magnification factors (40×, 100×, 200×, and 100×). ECSAnet demonstrated exceptional standalone performance, achieving its highest accuracy (99.52%) and F1-score (99.64%) at 40× magnification. At this level, ECSAnet leveraged global tissue-level features to attain perfect precision (100%) and the highest AUC (99.99%). At 100× magnification, ECSAnet maintained excellent performance, achieving an accuracy of 98.12% and its highest F1-score (98.66%), benefiting from the balance between global context and cellular details. At 200×, it achieved strong accuracy (98.07%) and F1-score (98.59%) by leveraging detailed cellular features, despite a slight increase in false positives and negatives. At 100×, where contextual information is limited due to the narrow field of view, ECSAnet delivered a strong F1-score (96.24%) and AUC (99.23%). However, its accuracy dropped to 94.71%, reflecting the challenges of classifying high-resolution cellular-level details.

For the hybrid approach, where ECSAnet served as a feature extractor for ML classifiers, the results demonstrated that several ML classifiers improved upon the standalone ECSAnet model’s performance, particularly at higher magnifications. At 40× and 100× magnifications, the ML classifiers maintained the same accuracy as the standalone ECSAnet model (99.52% and 98.12%, respectively), showing no additional benefit from hybridization at these levels. However, at 200× and 100×, several ML classifiers exceeded the standalone model’s performance. DT achieved the most improvements with accuracies of (99.52%) and (95.77%) at 200× and 100×. This shows that its ability to utilize ECSAnet’s extracted features aligns well with global and cellular structures at these resolutions. LR similarly improved upon the standalone model, matching DT’s accuracy of (99.52%) at 200× and achieving (95.24%) at 100×. Additionally, LDA and SVM improved performance at 200× (98.55%) and 100× (95.24%), while KNN only showed improvement.

From a magnification perspective, the results reveal the significant influence of resolution on performance. The strong performance at 40× aligns with the findings of Rashmi et al. [6], which recommend prioritizing low magnifications (40× and 100×) for breast cancer diagnosis due to their ability to provide an overview of tissue architecture and identify areas of interest. At 40×, the dominance of global tissue-level features enables the models to achieve high accuracy, while at 100×, the balance of global and cellular details allows ECSAnet and its hybrid configurations to maintain a high performance. At 200×, the dominance of cellular details enables DT and LR to excel, leveraging precise feature extraction. At 100×, the limited field of view reduces performance across classifiers; however, DT remains the most robust, handling fine-grained cellular details effectively. This decline may result from the challenges of analyzing cellular morphology at such high resolutions, where nuclear details (e.g., mitotic figures) become critical but are harder to standardize computationally. It is notable that as magnification increases, the performance of most models tends to decline slightly, particularly at 100× magnification. This trend can be attributed to the increased focus on fine-grained cellular details, which may introduce noise or irrelevant features for binary classification tasks.

In summary, ECSAnet performs exceptionally as a standalone model and feature extractor in hybrid configurations, with DT and LR outperforming other classifiers, particularly at 200× and 100× magnifications. The findings emphasize the importance of selecting appropriate magnification levels and classifier configurations to achieve optimal performance in breast histopathological image classification.

**Figure 8:**
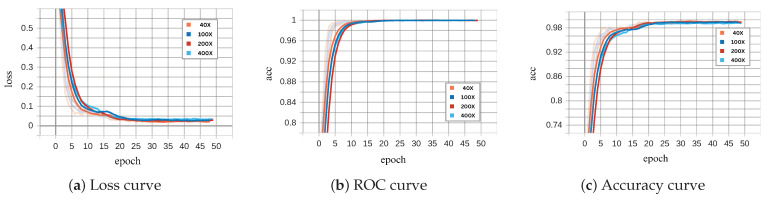
Binary ECSAnet performance on the training set across magnification factors.

**Figure 9:**
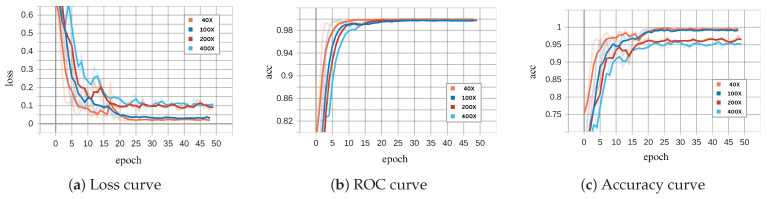
Binary ECSAnet performance on the validation set across magnification factors.

Figure 8 and Figure 9 illustrate the performance of the binary ECSAnet model on the training and validation sets across various magnification factors, respectively. These figures provide insights into the model’s adaptability, generalization ability, and robustness under different magnifications. The loss curves (subplots a) depict the optimization process, with both training and validation losses decreasing steadily over epochs. The training loss stabilizes near zero after approximately 25 epochs across all magnifications, reflecting effective convergence. Among the magnifications, 40× demonstrates the smoothest and most stable loss reduction, while higher magnifications, such as 100×, exhibit greater variability, likely due to the increased complexity of fine-grained cellular features. Similarly, the validation loss curves show a consistent decrease, with 40× and 100× magnifications achieving the lowest final loss values, further highlighting their superior generalization capabilities compared to 200× and 100× magnifications.

The ROC (subplots b) and accuracy curves (subplots c) further validate the model’s performance across magnifications. Training ROC curves approach near-perfect values (close to 1.0) within the first few epochs, particularly for 40× and 100× magnifications, underscoring the model’s strong discrimination ability. Validation ROC curves display a similar trend, with 40× and 100× achieving the most robust and consistent results, while higher magnifications, particularly 100×, show delayed convergence and greater variability. The accuracy curves reinforce these observations, with 40× magnification achieving the highest and most stable accuracy on both training and validation datasets. In contrast, higher magnifications (200× and 100×), while beneficial for cellular-level analysis, exhibit reduced performance due to increased noise and variability in smaller-scale features. These findings emphasize the diagnostic strengths of lower magnifications (40× and 100×) in providing a balance between tissue-level context and feature clarity, making them particularly effective for binary classification tasks.

Table 6, Table 7, Table 8 and Table 9 present the test set classification results of the ECSAnet model + DT for each breast tissue subtype across different magnification levels. The ECSAnet + DT model demonstrates exceptional performance in binary classification across different magnification levels, with near-perfect results at 40× and 200× magnifications. At these levels, the model achieves 99.52% accuracy, reflecting its ability to correctly classify both benign and malignant samples. Metrics such as precision, sensitivity, specificity, and F1-score for both classes are consistently high, with no significant discrepancies. This indicates that the model successfully generalizes across test samples and maintains robust decision boundaries. The balanced distribution of benign and malignant samples in these datasets likely contributes to the model’s consistent performance, ensuring equitable classification for both classes.

At 100× and 100× magnifications, the model’s accuracy drops slightly to 98% and 95.77%, respectively, indicating increased classification challenges. For the 100× test set, a minor decrease in specificity for malignant samples suggests that some benign samples are misclassified as malignant. At 100× magnification, the sensitivity for benign samples reduces to 90%, while the specificity for malignant samples drops to 90.16%, showing a slight tendency to confuse the two classes. These performance fluctuations could be attributed to increased feature variability (100×) or overlapping features and noise at higher magnifications (100×). Despite these challenges, the model maintains high F1-scores for both classes, reflecting its reliability in practical applications.

Overall, ECSAnet + DT achieves high accuracy and strong class-specific metrics across all magnification levels, with the best performance observed at 40× and 200×. The close alignment of macro and weighted averages highlights the model’s ability to handle class imbalance effectively, ensuring consistent results across the test set. While the performance of the model at 100× and 100× magnifications highlights areas for improvement, the model remains a reliable tool for distinguishing between benign and malignant samples. Future work could focus on addressing these performance variations through data augmentation or feature enhancement techniques tailored to specific magnifications.

### 4.2. Qualitative Results

**Figure 10:**
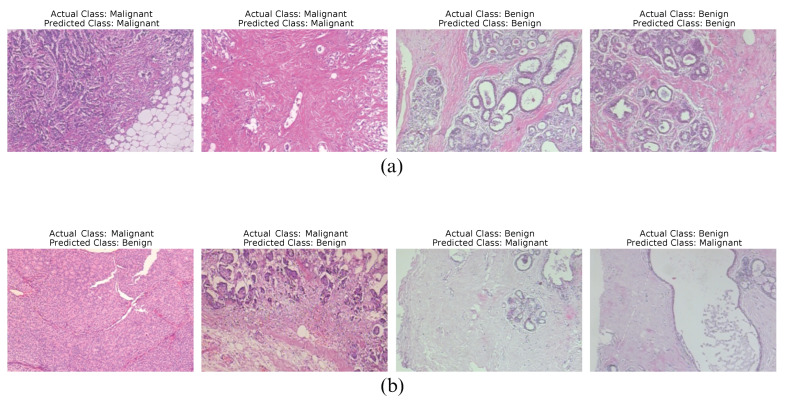
Sample results using ECSAnet + DT at 40× magnification: (**a**) displays examples of correctly classified images, while (**b**) presents examples of misclassified images.

As a qualitative analysis, Figure 10 provides a comprehensive overview using ECSAnet + DT at 40× magnification by showcasing samples of correctly classified images, which highlight the model’s accuracy, robustness, and effectiveness in capturing relevant features. Additionally, it includes samples of incorrectly classified images to emphasize the challenges, ambiguities, and limitations encountered during the classification process. These misclassifications provide valuable insights into potential sources of error, such as overlapping features between classes, noise in the data, or limitations in the model’s ability to generalize to certain patterns. By analyzing both correct and incorrect classifications, this figure not only underscores the strengths of the model but also serves as a diagnostic tool to identify areas for improvement, such as refining feature extraction techniques, optimizing model parameters, or integrating additional modalities to enhance performance. This dual perspective contributes to a more nuanced understanding of the model’s behavior and its applicability in real-world scenarios.

### 4.3. Comparative Analysis with the Literature

**Figure 11:**
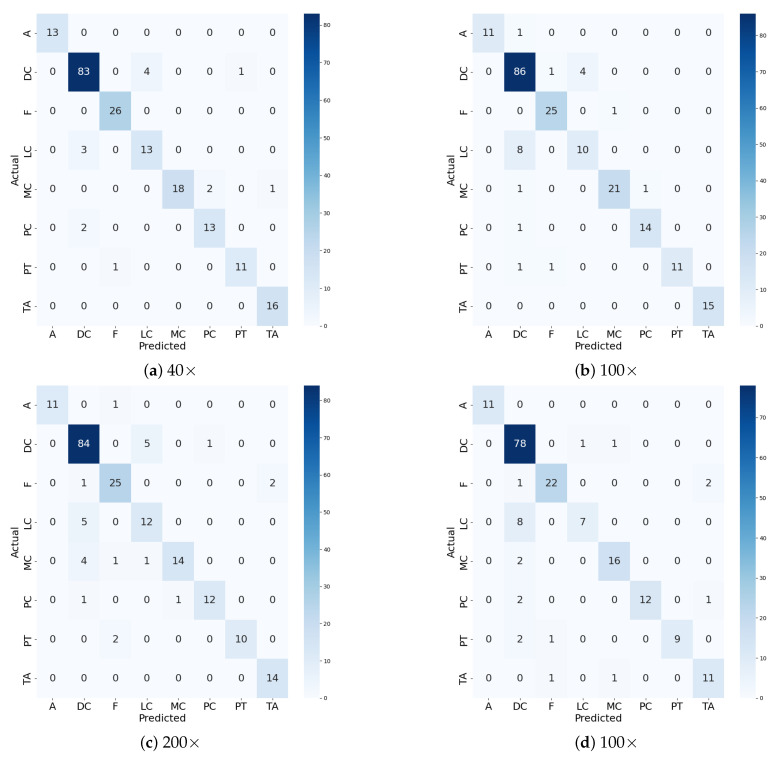
Confusion matrices for ECSAnet+DT for multi-class classification: benign and malignant across magnification factors [5].

**Figure 12:**
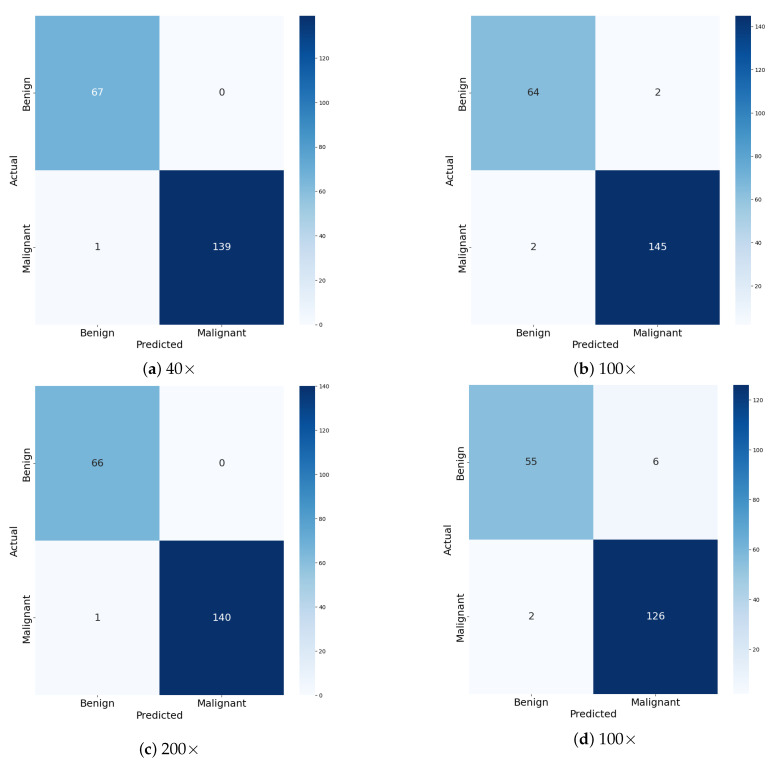
Confusion matrices for ECSAnet + DT for binary classification: benign and malignant across magnification factors.

The confusion matrices in Figure 11 and Figure 12 highlight the performance of ECSAnet + DT for both multi-class and binary classification tasks across different magnification levels (40×, 100×, 200×, and 100×). For multi-class classification, lower magnifications (40× and 100×) demonstrate superior performance, with most predictions correctly aligned along the diagonal, indicating accurate differentiation between subtypes. However, as magnification increases to 200× and 100×, off-diagonal misclassifications become more frequent, reflecting greater difficulty in distinguishing between subtypes due to the focus on fine-grained details, which may obscure broader tissue-level patterns. This suggests that lower magnifications provide a more comprehensive view of tissue structures, aiding in accurate multi-class classification.

Binary classification, on the other hand, achieves high accuracy across all magnifications, with fewer misclassifications compared to the multi-class task. At 40× and 200×, the model achieves near-perfect results, with minimal false positives and false negatives. While the model maintains strong performance at 100× and 100×, there is a slight increase in misclassifications. The reduced complexity of binary classification, which only requires distinguishing between benign and malignant samples, makes it less sensitive to noise introduced at higher magnifications.

Overall, the results emphasize the diagnostic strength of lower magnifications (40×, 100×) for both tasks, as they capture tissue-level context and spatial patterns more effectively, leading to higher accuracy and robustness. High magnifications, while valuable for cellular-level analysis, introduce variability and noise that can hinder performance, particularly for multi-class classification. These findings underscore the importance of tailoring magnification levels to the classification objective, with lower magnifications being particularly advantageous for tasks requiring broader structural context.

**Figure 13:**
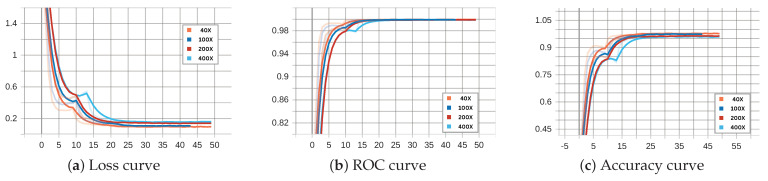
Multi-class ECSAnet performance on the training set across magnification factors.

**Figure 14:**
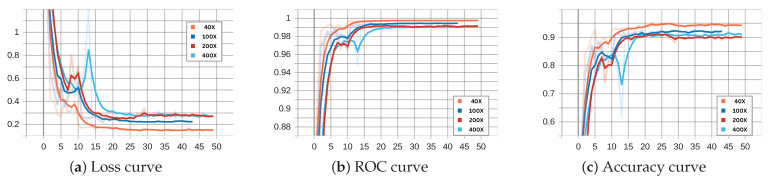
Multi-class ECSAnet performance on the validation set across magnification factors.

The charts in Figure 8 and Figure 9 (binary classification) and Figure 13 and Figure 14 (multi-class classification) reveal distinct behaviors during training and validation across magnifications. Binary classification achieves faster loss reduction and lower final loss values, reflecting its simpler nature compared to multi-class classification. In multi-class tasks, higher magnification (200× and 100×) leads to higher validation loss due to increased complexity and noise, while 40× and 100× perform more robustly by leveraging tissue-level context. The ROC and accuracy curves further emphasize these trends.

Binary classification achieves near-perfect ROC values and higher accuracy across all magnifications, with 40× and 100× consistently outperforming higher magnifications. Multi-class classification shows greater variability, especially at 100×, where fine-grained features introduce noise that complicates subtype differentiation. Overall, 40× and 100× magnifications balance feature clarity and context effectively, making them superior for both tasks, while higher magnifications are less reliable due to increased noise and complexity.

## 5. Discussion of Results

### 5.1. Main Findings

The results underscore the importance of selecting the appropriate magnification level and classification model for specific diagnostic objectives:

- Low Magnifications (40×, 100×, 200×): These levels provide a broader view of tissue architecture, making them valuable for identifying larger structures or patterns. The high performance at these magnifications supports their use as the primary diagnostic focus in clinical practice.
- High Magnification (100×): This level is critical for detailed cellular analysis, such as assessing nuclear atypia and mitotic figures, but it introduces challenges such as increased noise and feature variability. While high magnifications are essential for specific diagnostic tasks, their utility in binary classification may be limited compared to lower magnifications.

### 5.2. Model Strengths

- High Accuracy Across All Magnifications: The majority of predictions for both benign and malignant classes are correctly classified, as evident from the strong diagonal dominance in the confusion matrices (e.g., 87/88 correctly classified benign samples at 40× and 345/347 malignant samples at 100×). This demonstrates the model’s ability to generalize well across magnification levels.
- Low False Negative Rate: The number of malignant cases misclassified as benign (false negatives) is minimal (e.g., 1 false negative at 40× and only 1–2 across other magnifications). This is critical in medical diagnostics to minimize missed cancer diagnoses.
- Robust Performance at Lower Magnifications (40×, 100×, and 200×): At 40×, 100×, and 200×, the model achieves high classification accuracy with minimal errors, showing its strength in leveraging broader tissue-level contexts for binary classification tasks.

### 5.3. Model Limitations

- Slight Increase in Errors at High Magnification (100×): At 100×, the number of false positives (benign samples misclassified as malignant) slightly increases (e.g., eight at 100× compared to seven at 100×). This suggests that higher magnifications introduce noise and fine-grained details that might confuse the model. Similar observations have been made in other studies, such as the binary classification work by Li et al. [25] and the multi-class classification research by Boumaraf et al. [26]. Conversely, some studies, including [27], reported reduced classification performance at the 100× magnification level. Taheri et al. [28] provides more insights into how magnification and classification type impact model performance, aiming to improve the accuracy and reliability of automated cancer diagnosis systems.
- Imbalance in Error Distribution: False positives (benign misclassified as malignant) are slightly more common than false negatives, which could lead to unnecessary follow-up procedures. While this is less critical than false negatives, it still reflects a slight bias in the model. Clement et al. [29] emphasizes strategies like class balancing, the careful selection of features across resolutions, and the use of evaluation metrics that account for imbalances.
- Sensitivity to Magnification Variability: Although the model performs well overall, the slight drop in performance at higher magnifications indicates a limitation in consistently handling fine-grained cellular details where contextual information is reduced.

## 6. Conclusions and Future Work

This study highlights the importance of tailoring magnification levels to specific diagnostic objectives in breast cancer classification. Lower magnifications (40×, 100×) consistently outperformed high magnification (200×, 100×) in accuracy, robustness, and generalization, particularly in multi-class classification. Lower magnifications provided a broader tissue-level context, enabling a better differentiation of major types, while higher magnifications introduced noise and variability due to fine-grained details. Binary classification demonstrated strong performance across all magnifications but benefitted most from the stability of lower magnifications.

In the future, to further enhance diagnostic accuracy and robustness, we propose the following strategies:

- Multimodal Imaging: Combining sMRI, rs-fMRI, or other imaging modalities could provide complementary information and enhance model performance.
- Hybrid Architectures: While this paper demonstrates the effectiveness of combining CNNs with ensemble classifiers such as DT and SVM, future work will explore further optimization and evaluation of these hybrid architectures. This includes refining integration techniques, assessing their performance on more complex datasets, and identifying scenarios where such combinations are most beneficial for feature extraction and classification.
- Magnification-Specific Models: Developing specialized models optimized for specific magnification might address the limitations observed at higher resolutions.

In summary, the results showed the effectiveness of integrating ML classifiers with DL models, creating hybrid architectures for the binary classification of BC histopathological images, and, in particular, increasing DL model accuracy at higher magnifications, which highlights the critical role of magnification levels and classifier selection in histopathological image analysis. These findings pave the way for developing more effective, multimodal, and scalable diagnostic frameworks. The clinical applicability of our approach holds significant potential. By improving diagnostic accuracy at specific magnification levels, it can assist pathologists in making more precise diagnoses, supporting early detection and reducing the risk of human error. The scalable hybrid architecture can be adapted to various tasks and integrated into clinical systems, streamlining workflows and enhancing decision-making in complex cases. This approach addresses key challenges in histopathological analysis, contributing to more efficient and accurate cancer diagnostics.

## Figures and Tables

**Table 1:** Class distribution of BreakHis dataset.

Magnification	Benign				Malignant				Total
	A	F	PT	TA	DC	LC	MC	PC	
40×	114	253	109	149	864	156	205	145	1995
100×	113	260	121	150	903	170	222	142	2081
200×	111	264	108	140	896	163	196	135	2013
100×	106	237	115	130	788	137	169	138	1820
**Subtype total**	444	1014	453	569	3451	626	792	560	7909
**Type total**	2480				5429				7909

**Table 2:** Class distribution of the training set in the BreakHis dataset before and after balancing and oversampling.

Before Balancing and Oversampling									
**Magnification**	**Benign**				**Malignant**				**Total**
	**A**	**F**	**PT**	**TA**	**DC**	**LC**	**MC**	**PC**	
40×	80	177	76	104	603	109	143	101	1393
100×	79	181	85	105	631	119	155	99	1454
200×	77	184	75	98	626	114	137	94	1405
100×	74	165	80	91	550	97	118	96	1271
**Subtype total**	310	707	316	398	2410	439	553	390	5523
**Type total**	1731				3792				5523
**After balancing and oversampling**									
40×	1812	1812	1812	1812	1812	1812	1812	1812	14,496
100×	1896	1896	1896	1896	1896	1896	1896	1896	15,168
200×	1881	1881	1881	1881	1881	1881	1881	1881	15,048
100×	1653	1653	1653	1653	1653	1653	1653	1653	13,224
**Subtype total**	7242	7242	7242	7242	7242	7242	7242	7242	57,936
**Type total**	28,968				28,968				57,936

**Table 3:** A summary of the statistical performance metrics used for model comparisons.

Metrics	Formula	Definition
Accuracy	$\frac{TP+TN}{TP+TN+FP+FN}(*)$	This is the ratio of correctly predicted instances (true positives and true negatives) to the total number of instances in the dataset, measuring the model’s overall correctness.
Precision	$\frac{TP}{TP+FP}$	This is the ratio of true positive predictions to the total number of positive predictions made by the model, indicating the model’s accuracy for the positive class.
Recall	$\frac{TP}{TP+FN}$	This is the ratio of true positive predictions to the total actual positives in the dataset, measuring the model’s ability to identify all relevant instances of the positive class.
Jaccard index	$\frac{TP}{TP+FN+FP}$	Also known as the Intersection over Union (IoU), this measures the similarity between the predicted and ground truth sets by calculating the size of their intersection divided by their union.
F1-score	$\frac{2\;\cdot \;TP}{2\;\cdot \;TP+FP+FN}$	This is the harmonic mean of Precision and Recall, providing a balanced measure of a model’s accuracy when Precision and Recall are of different magnitudes.
Matthews Correlation Coefficient (MCC)	$\frac{TP\;\cdot \;TN-FP\;\cdot \;FN}{\sqrt{\left(TP+FP)(TP+FN)(TN+FP)(TN+FN\right)}}$	This is a performance metric that evaluates the quality of binary classifications, and is particularly useful for imbalanced datasets as it considers all four confusion matrix components (TP, TN, FP, FN).

* Where TP stands for true positives, TN for true negatives, FP for false positives, and FN for false negatives.

**Table 4:** The hyperparameter values used for training in this study.

Hyperparameter	Value
Learning Rate	0.001
Learning Rate Scheduler	Reduce LROnPlateau
Early Stopping	Patience = 25 epochs
Weight Decay	0.01
Number of Epochs	50
Batch Size	16
Optimizer	SGD
Loss Function	CrossEntropyLoss

**Table 5:** Binary ECSAnet performance metrics across magnification factors.

Model	Magnification	Accuracy (%)	Precision (%)	F1-Score (%)	Jaccard Index (%)	AUC (%)	MCC (%)
ECSAnet [5]	40×	**99.52**	**100.00**	**99.64**	**99.29**	**99.99**	99.00
	100×	**98.12**	97.35	**98.66**	**97.35**	**99.59**	96.00
	200×	98.07	97.90	98.59	97.22	**99.94**	95.00
	100×	94.71	92.75	**96.24**	**92.75**	**99.23**	86.00
ECSAnet [5] + DT	40×	**99.52**	99.52	99.52	99.04	99.64	99.00
	100×	**98.12**	**98.12**	98.12	96.32	97.80	96.00
	200×	**99.52**	**99.52**	**99.52**	**99.04**	99.65	99.00
	100×	**95.77**	**95.79**	95.73	91.86	94.30	90.24
ECSAnet [5] + KNN	40×	**99.52**	99.52	99.52	99.04	99.64	99.00
	100×	**98.12**	**98.12**	98.12	96.32	97.79	96.00
	200×	98.07	98.07	98.06	96.20	99.63	98.00
	100×	95.24	95.39	95.16	90.83	95.66	91.00
ECSAnet [5] + LDA	40×	**99.52**	99.52	99.52	99.04	99.64	99.00
	100×	**98.12**	**98.12**	98.12	96.32	97.80	96.00
	200×	98.55	98.55	98.55	97.14	98.47	98.00
	100×	95.24	95.28	95.18	90.87	93.81	91.00
ECSAnet [5] + LR	40×	**99.52**	99.52	99.52	99.04	**99.99**	99.00
	100×	97.65	97.65	97.65	95.42	99.59	96.00
	200×	**99.52**	**99.52**	**99.52**	**99.04**	99.89	99.00
	100×	95.24	95.28	95.18	90.87	**99.23**	91.00
ECSAnet [5] + NB	40×	98.07	98.18	98.08	96.24	98.57	98.00
	100×	96.24	96.38	96.27	92.85	96.44	94.00
	200×	98.07	98.07	98.06	96.20	97.37	98.00
	100×	94.71	94.89	94.75	90.08	94.81	84.00
ECSAnet [5] + SVM	40×	**99.52**	99.52	99.52	99.04	**99.99**	99.00
	100×	**98.12**	**98.12**	98.12	96.32	97.55	96.00
	200×	98.55	98.55	98.55	97.14	99.92	98.00
	100×	95.24	95.39	95.16	90.83	95.47	91.00

Note: Bold fonts indicate the best values.

**Table 6:** ECSAnet + DT binary classification results for each class on the 40× magnification test set.

Class	Precision (%)	Sensitivity (%)	Specificity (%)	F1-Score (%)	Support
Benign	99.00	100.00	99.29	99.00	62
Malignant	100.00	99.00	100.00	100.00	140
Accuracy				100.00	207
Macro Average	99.00	100.00	99.64	99.00	207
Weighted Average	100.00	100.00	99.76	100.00	207

**Table 7:** ECSAnet + DT binary classification results for each class on the 100× magnification test set.

Class	Precision (%)	Sensitivity (%)	Specificity (%)	F1-Score (%)	Support
Benign	97.00	97.00	98.64	97.00	66
Malignant	99.00	99.00	96.97	99.00	147
Accuracy				98.00	213
Macro Average	98.00	98.00	97.80	98.00	213
Weighted Average	98.00	98.00	97.52	98.00	213

**Table 8:** ECSAnet + DT binary classification results for each class on the 200× magnification test set.

Class	Precision (%)	Sensitivity (%)	Specificity (%)	F1-Score (%)	Support
Benign	99.00	100.00	99.29	99.00	66
Malignant	100.00	99.00	100.00	100.00	141
Accuracy				100.00	207
Macro Average	99.00	100.00	99.64	99.00	207
Weighted Average	100.00	100.00	99.76	100.00	207

**Table 9:** ECSAnet + DT binary classification results for each class on the 100× magnification test set.

Class	Precision (%)	Sensitivity (%)	Specificity (%)	F1-Score (%)	Support
Benign	96.00	90.00	98.44	93.00	61
Malignant	95.00	98.00	90.16	97.00	128
Accuracy				96.00	189
Macro Average	96.00	94.00	94.30	95.00	189
Weighted Average	96.00	96.00	92.83	96.00	189

## Data Availability

The raw data supporting the conclusions of this article will be made available by the authors on request.
